# Effects of hydrocortisone and yohimbine on selective attention to emotional cues

**DOI:** 10.1177/0269881121997100

**Published:** 2021-03-28

**Authors:** Sophie Metz, Woo R Chae, Christian E Deuter, Christian Otte, Katja Wingenfeld

**Affiliations:** Klinik und Hochschulambulanz für Psychiatrie und Psychotherapie, Charité – Universitätsmedizin Berlin, Corporate Member of Freie Universität Berlin, Humboldt-Universität zu Berlin, and Berlin Institute of Health, Berlin, Germany

**Keywords:** Stress, hydrocortisone, yohimbine, attentional bias, dot-probe task

## Abstract

**Introduction:**

Facial expressions contain important affective information, and selective attention to facial expression provides an advantage in the face of loss, stress and danger. In addition, the sympathetic nervous system and hypothalamus-pituitary-adrenal axis mediate the organism’s response to loss and danger. Here, we aimed at investigating the influence of sympathetic nervous system and hypothalamus-pituitary-adrenal axis activation on selective attention to affective facial stimuli.

**Methods and materials:**

One hundred-and-four healthy men between 18–35 years old (mean (standard deviation) age: 24.1 (3.5) years) participated in the study. We used a randomised, double-blind, placebo-controlled design. Participants received either: (a) yohimbine, (b) hydrocortisone, (c) yohimbine and hydrocortisone or (d) placebo only and participated in a dot-probe task with sad, happy and neutral faces. We collected salivary samples to measure cortisol and alpha amylase activity in addition to measurements of blood pressure and heart rate. Salivary cortisol served as correlate of hypothalamus-pituitary-adrenal axis activation and salivary alpha amylase activity, blood pressure and heart rate as correlates of sympathetic nervous system activation. Measurements were carried out before and after drug administration.

**Results:**

We did not find a main effect or interaction effect of hydrocortisone or yohimbine administration on selective attention to happy faces. However, we found an interaction of yohimbine and hydrocortisone on selective attention to sad faces. Post-hoc *t*-test revealed an attentional bias away from sad stimuli and towards neutral faces in the hydrocortisone-only group.

**Discussion:**

Only hydrocortisone administration led to an attentional bias away from sad faces. Future studies should investigate these effects in major depression disorder, as this disorder is characterised by glucocorticoid resistance and increased processing of sad stimuli.

## Introduction

As facial expressions contain important affective information, selective attention to facial expression provides an advantage in the face of loss and danger. The sympathetic nervous system (SNS) and hypothalamus-pituitary-adrenal (HPA) axis mediate the organism’s response, for example to danger (Vasa et al., 2009). We aimed to further characterise the influence of SNS and HPA axis activation on selective attention.

Exogenous cortisol reduced selective attention to threat-related stimuli (Putman et al., 2007) and sadness-induced subgenual cingulate activity (Sudheimer et al., 2013). Exogenous cortisol binds to the glucocorticoid receptor (GR) and mineralocorticoid receptor (MR) and thereby possibly influences activation in emotion-related brain regions.

Selective attention towards sad faces after the administration of fludrocortisone, a MR agonist, has also been shown (Schultebraucks et al., 2016). MR effects and activation of the noradrenergic system in the early phase of the stress response might explain effects in the fludrocortisone condition. This is in line with a study investigating SNS activation reporting selective attention to salient stimuli after arousal induction (Lee et al., 2014). These results suggest that early MR effects and SNS activation enable fast reactions to negative facial expressions such as sadness, disgust, fear and anger with enhanced attentional bias, while GR activation has opposing effects, i.e. a reduced attentional bias.

A study with combined SNS and HPA axis activation revealed an attentional bias toward threat-related stimuli in the placebo group, but not after yohimbine or hydrocortisone administration, nor its combination (Vasa et al., 2009). Similar results have been shown with psychosocial stress induction (Jiang et al., 2017). However, another study reported an attentional bias towards negative emotional stimuli after psychosocial stress (Roelofs et al., 2007).

Taken together, these results suggest that while a cortisol increase attenuates attentional bias, SNS activation enables attentional bias. Results of combined HPA axis and SNS activation reveal heterogeneous results. The aim of the current study was to investigate the influence of HPA axis and SNS activation on selective attention to happy and sad stimuli using a dot-probe paradigm. As there are no studies investigating these effects on sad stimuli and depression is primarily associated with glucocorticoid resistance (Pariante, 2017) and an attentional bias to sad faces (Dai et al., 2016), an attentional bias to sad faces in association with HPA axis and SNS activation is worthwhile investigating.

We expected to find a decrease in an attentional bias toward sad faces after hydrocortisone administration. Furthermore, we expected to find an increased attentional bias towards sad faces after yohimbine administration. We investigated combined hydrocortisone and yohimbine administration in an exploratory manner. We expected to find no effect of treatment on attentional bias toward happy faces as they do not imply distress.

## Materials and methods

### Participants

One hundred-and-four healthy men between 18–35 years old (mean (standard deviation (SD)) age: 24.1 (3.5) years) participated in the study. As menstrual cycle or contraception might affect circulating cortisol (Hamidovic et al., 2020) and basal and stimulated cortisol is altered in young and old age (Gunnar et al., 2009; Nater et al., 2013), we decided to only include male participants between 18–35 years old. All participants had a body mass index <30, were native German speakers and were not taking any medication. Central nervous system or somatic diseases, metabolic or endocrine diseases, current infection or autoimmune diseases, and current or past psychiatric disorders were exclusion criteria. Participants signed a written consent form, and the local ethics committee approved the study. They received compensation of 60€ to 80€ depending on their payoff in a task presented elsewhere (Metz et al., 2020). The study took place at the Department of Psychiatry and Psychotherapy, Charité – Universitätsmedizin Berlin, Campus Benjamin Franklin, Germany. Further details about exclusion criteria and sample characteristics can be found elsewhere (Chae et al., 2019).

### Procedure

We used a randomised, double-blind, placebo-controlled design. Participants were assigned to one of four groups: (a) yohimbine+placebo (10 mg, *n=*26), (b) placebo+hydrocortisone (10 mg, *n=*26), (c) yohimbine+hydrocortisone (10 mg each, *n=*26), or (d) placebo+placebo (*n=*26). All experimental sessions took place in the afternoon. Yohimbine was given 75 min and hydrocortisone 60 min before the task started. In earlier studies, we and others used yohimbine (Deuter et al., 2020) and hydrocortisone (Kirschbaum et al., 1996; Terfehr et al., 2011; Tops et al., 2003) with similar doses and timing as proxies of the SNS and HPA axis respectively, and successfully revealed an increase in salivary alpha-amylase (sAA) activity and cortisol.

Blood pressure and heart rate were measured, and saliva samples were collected to measure salivary cortisol and sAA activity. Samples were collected at two baseline measurements (+0 min and +15 min) and at three time-points after drug administration (+75 min (before dot-probe task), +105, +135 min (after dot-probe task). Further details can be found in Chae et al. (2019).

The dot-probe task was part of a more extensive study (Chae et al., 2019; Metz et al., 2020). Task order was always the same; the dot-probe task was the second task and took ∼12 min to complete.

#### Task

**Figure 1.**
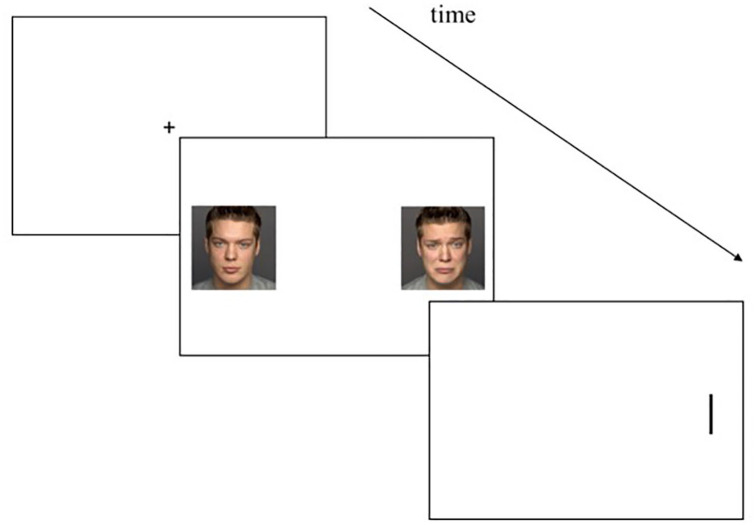
Schematic representation of the dot-probe task. Sad congruent trial: the cue replaces the sad emotional facial expression.

To measure selective attention toward emotional cues, we used a dot-probe paradigm similar to Schultebraucks et al. (2016). Stimuli were human faces from the FACES database (Ebner et al., 2010). Pictures of 20 persons (10 female and 10 male) with happy, sad, and neutral facial expressions were taken from that set. Each trial started with a fixation cross (500 ms). Subsequently, two pictures of human faces appeared on the screen (500 ms). The two pictures displayed two facial expressions of the same person, with one expression displayed on the left-hand side and one expression on the right-hand side of the screen. Pairs included a combination of neutral-sad, neutral-happy, or neutral-neutral facial expression. A vertical bar as cue (1100 ms) replaced the left or the right picture. The participants were instructed to respond as quickly as possible to the position of the cue (right vs left) and to press the right or left key accordingly. Response latency reflect attentional capture. Participants ought to react quicker to the cue when it replaces the picture that antecedently attracted their attention. If the cue replaces the emotional stimulus, the condition is called ‘congruent’ (see Figure 1). If the cue replaces the neutral stimuli, the condition is called ‘incongruent’. The neutral-neutral condition was used as control condition. Each participant completed 200 trials, i.e. 40 trials in the neutral condition, 40 trials in the happy congruent condition, 40 trials in the happy incongruent condition, 40 trials in the sad congruent condition and 40 trials in the sad incongruent condition. Order between all trials was quasi-randomised, and position of pictures was counterbalanced (left vs right).

An ‘attentional bias index’ (AB) is determined by subtracting the average reaction times of congruent trials from the average reaction times of incongruent trials (AB=1/2*((incongruence right − congruence right)+(incongruence left − congruence left))). A positive AB reflects an attentional bias toward the emotional stimulus, and a negative AB an attentional bias toward the neutral stimulus (i.e. avoiding the emotional stimulus). For further details, see Schultebraucks et al. (2016).

### Data analysis

Data were unavailable for three participants due to technical problems. Before calculating AB, we excluded reaction times that were less than 100 ms (anticipation error) and greater than 1500 ms (lack of concentration). We only analysed response latencies of correct responses. We excluded two outliers (<3 SD) in AB of sad and happy stimuli. Ninety-nine participants were included in the final analysis (yohimbine+placebo, *n*=23; hydrocortisone+placebo, *n*=26; yohimbine+hydrocortisone, *n*=25; placebo+placebo, *n*=25).

Statistical analysis was performed using SPSS version 25. AB values were analysed using multivariate analysis of variance (MANOVA), with the dependent variables AB for sad and AB for happy stimuli, and the between-subject factors yohimbine (yes/no) and hydrocortisone (yes/no). Furthermore, separate analyses of variance (ANOVAs) of AB for sad and AB for happy stimuli were performed, followed by post-hoc *t*-tests to compare treatment groups.

We examined AB within treatment groups using one-sample *t*-tests to reveal whether AB differed from zero. This served to reveal whether there was an AB in the placebo group and any treatment group.

Analysis of saliva samples, blood pressure, heart rate and sample characteristics are described elsewhere (Chae et al., 2019; Metz et al., 2020). However, we calculated Baseline to Peak (BtP) (maximum value of measurement point 3 to 5 minus mean of measurement point 1 and 2) of cortisol, sAA activity, blood pressure and heart rate (see Supplementary Material Table S1).

We additionally correlated BtP of cortisol, sAA activity, blood pressure and heart rate with AB of sad and happy faces.

## Results

### Sample characteristics and treatment check

Results on sample characteristics and on treatment check variables are presented elsewhere (Chae et al., 2019; Metz et al., 2020). Briefly, there were no significant differences in demographics (apart from BMI) between treatment groups (see Supplementary Material Table S1). Obese participants were excluded and the number of overweight participants did not differ between groups. Hydrocortisone significantly increased saliva cortisol and systolic blood pressure. Given the increase in saliva cortisol and systolic blood pressure we assume the HPA axis has been activated. Yohimbine significantly increased systolic and diastolic blood pressure and sAA activity. Given increased systolic and diastolic blood pressure and sAA activity we assume the SNS has been activated. No treatment increased heart rate (see Supplementary Material Table S1).

### Dot-probe task

MANOVA with the dependent variables AB for sad stimuli and AB for happy stimuli did not show a main effect of yohimbine (*F*(2,94)=0.89, *p*=0.41, *η*^2^ =0.02) or hydrocortisone (*F*(2,94)=0.17, *p*=0.85, *η*^2^=0.00). However, an interaction effect of yohimbine and hydrocortisone on AB became significant (*F*(2,94)=3.17, *p* <0.05, *η*^2^=0.06).

Post-hoc ANOVA on AB for happy stimuli revealed neither a main effect of yohimbine (*F*(1,95)=0.15, *p*=0.70, *η*^2^=0.00) and hydrocortisone (*F*(1,95)=0.13, *p*=0.72, *η*^2^=0.00) nor an interaction of both (*F*(1,95)=0.65, *p*=0.42, *η*^2^=0.01).

**Figure 2.**
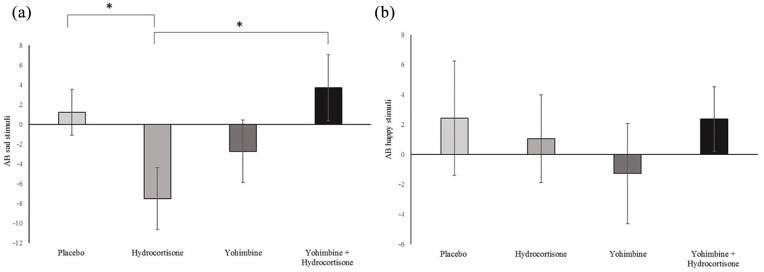
Attentional bias: sad and happy stimuli. A positive attentional bias (AB) score indicates an AB toward the sad (a) or happy (b) stimuli and a negative score an AB toward the neutral stimulus. *p <0.05.

Concerning AB for sad stimuli, no main effect of yohimbine (*F*(1,95)=1.45, *p*=0.23, *η*^2^=0.02) and hydrocortisone (*F*(1,95)=0.15, *p*=0.71, *η*^2^=0.00) were revealed. However, an interaction of yohimbine with hydrocortisone became significant (*F*(1,95)=6.28, *p*=0.01, *η*^2^=0.06). Post-hoc *t*-test revealed that AB for sad emotional stimuli in the hydrocortisone only group differed from the combined group (*t*(49)=2.44, *p*=0.02) and the placebo group (*t*(49)=−2.22, *p*=0.03) (see Figure 2). This suggests an AB away from sad faces only after hydrocortisone administration.

We used one-sample *t*-tests to reveal differences from zero and found an AB away from sad emotional stimuli in the hydrocortisone only group (*t*(25)=−2.40, *p*=0.02), but not in any other treatment group.

No correlation between any BtP measurements and AB for sad or happy stimuli could be revealed (*p*>0.05).

## Discussion

In line with our hypothesis, we did not find an effect of hydrocortisone or yohimbine or its combination on selective attention to happy faces. Furthermore, we found no main effect of yohimbine and hydrocortisone on selective attention to sad faces. However, an interaction of yohimbine with hydrocortisone became significant with an increase in an attentional bias away from sad emotional stimuli in the hydrocortisone only group differing from the combined group and the placebo group. Our results suggest an attentional bias away from sad stimuli after hydrocortisone administration but in no other treatment group. This in line with our hypothesis of a decrease in an attentional bias toward sad faces after hydrocortisone administration. The results, however, contradict our hypothesis of an increased attentional bias after yohimbine administration.

Selective attention towards neutral stimuli (away from sad stimuli) increased after hydrocortisone administration. This is in line with the finding that cortisol reduces selective attention to threat (Putman et al., 2007). Furthermore, it has been shown that cortisol reduced sadness-induced subgenual cingulate activity (Sudheimer et al., 2013). Cortisol might affect sadness induced activation in related brain regions and thereby attenuate selective attention to sad faces. These effects also await to be determined in depression, which is associated with subgenual cingulate hyperactivity and glucocorticoid resistance (Sudheimer et al., 2013). Contradicting results with no bias after hydrocortisone administration or stress induction (Jiang et al., 2017; Vasa et al., 2009) or an increased attentional bias toward negative stimuli after stress induction have also been described (Roelofs et al., 2007).

We did not find an effect of yohimbine on selective attention, contradicting our hypothesis. Selective attention to salient stimulus after arousal induction in healthy participants has been described (Lee et al., 2014). As we used a pharmacological approach no arousal was induced, which of course is an important component of the stress response. Additionally, we used non-arousing negative stimuli, namely sad faces instead of angry or fearful faces. One might speculate that arousal is required to induce AB. In a former study, we revealed an attentional bias away from sad faces in the placebo condition and a shift towards sad faces after fludrocortisone administration (Schultebraucks et al., 2016). It might be the case that an increased attentional bias towards negative emotional stimuli is to be found in the early phase of the stress response, with both noradrenergic system and rapid MR activation.

Our missing results concerning selective attention to happy faces are in line with our former study (Schultebraucks et al., 2016). In addition, as stress mediates the organism’s response to e.g. loss and danger, we assume that selective attention to especially negative facial expressions is influenced by stress. However, results concerning selective attention towards positive emotional faces are scarce.

A strength of this study is that different stress neuromodulators were investigated. However, since pharmacological manipulation differs from the physiological stress response, results are difficult to compare to studies using arousal induction or different stressors. We only tested male participants between and 18–35 years old, so limited inference can be drawn regarding the same process in women or different age groups. Although many previous studies have investigated the effect of stress on threat-related stimuli, studies investigating the effect of separate HPA axis and SNS activation on sad and happy emotional stimuli in healthy participants are scarce. Thus, replication and studies on other non-arousing negative emotions such as fatigue are needed. Yohimbine was given 75 min before the task started. It is therefore possible that acute noradrenergic activation was missed, thus explaining our missing results concerning yohimbine.

Taken together, hydrocortisone administration decreased selective attention toward negative emotional cues, and more precisely increased attention toward neutral cues. Cortisol might have inhibitory effects on attention to emotional stimuli and attenuate selective attention to especially sad faces. Future studies should investigate these effects in depression as an attentional bias to sad faces and glucocorticoid resistance have been shown in these patients.

## Supplemental Material

sj-docx-1-jop-10.1177_0269881121997100 – Supplemental material for Effects of hydrocortisone and yohimbine on selective attention to emotional cues Click here for additional data file. Supplemental material, sj-docx-1-jop-10.1177_0269881121997100 for Effects of hydrocortisone and yohimbine on selective attention to emotional cues by Sophie Metz, Woo R Chae, Christian E Deuter, Christian Otte and Katja Wingenfeld in Journal of Psychopharmacology
