# Supplementary material for: Effects of hydrocortisone and yohimbine on selective attention to emotional cues
Source: J Psychopharmacol. 2021 Mar 28;35(6):755–9. doi: 10.1177/0269881121997100 (PMC8278549; doi:10.1177/0269881121997100)
Supplement: sj-docx-1-jop-10.1177_0269881121997100 – Supplemental material for Effects of hydrocortisone and yohimbine on selective attention to emotional cues [file sj-docx-1-jop-10.1177_0269881121997100.docx]

**Table S1.** Demographic data.

| Variable (M, SD) | Placebo | Yohimbine | Hydro-cortisone | Yohimbine & Hydro-cortisone | Statistics | *p* |
| --- | --- | --- | --- | --- | --- | --- |
| Age, years | 23.81 (3.36) | 23.19 (3.29) | 24.54 (4.04) | 24.69 (3.44) | *F*(3,100)=0.10  *η^2^* = 0.3 | .40 |
| Smoker | 2 | 4 | 4 | 5 | *χ²*(3) = 1.48  *V* = .20 | .69 |
| Years of  education | 12.88 (.59) | 12.65 (.98) | 12.88 (.59) | 12.85 (.78) | *F*(3,100)=0.56 *η^2^* = 0.2 | .64 |
| BMI (kg/m^2^) | 21.92 (1.83) | 23.91 (2.63) | 23.81 (2.88) | 23.46 (1.81) | *F*(3,100)=4.07 *η^2^* = 0.11 | .01 |
| BtP sAA activity (nmol/l) | 42.42 (62.01) | 73.05 (71.15) | 37.28 (57.01) | 84.66 (81.32) | F(3,100)=2.96  *η^2^* = 0.08 | .03 |
| BtP Cortisol (nmol/l) | -0.72 (2.12) | 2.03 (7.47) | 29.61 (25.47) | 26.72 (15.56) | F(3,100)=26.56  *η^2^* = 0.45 | .00* |
| BtP  heart rate (bpm) | -4.98 (5.91) | -1.52 (10.21) | -4.01 (6.82) | 2.13 (19.67) | F(3,100)=1.83  *η^2^* = 0.05 | .15 |
| BtP systolic blood pressure  (mmHg) | 1.52 (7.72) | 7.50 (8.55) | -0.10 (8.90) | 9.65 (11.01) | F(3,100)=6.81  *η^2^* = 0.17 | .00** |
| BtP diastolic blood pressure  (mmHg) | 2.03 (5.06) | 3.90 (5.20) | 2.15 (5.70) | 6.96 (6.27) | F(3,100)=4.41*η^2^* = 0.12 | .01*** |

BMI = Body Mass Index, BtP = Baseline to Peak (max(t3,t4,t5)-mean(t1,t2)), sAA = salvary alpha amylase. Results based on Chae et al. (2019) and Metz et al (2020).

* Bonferroni corrected post-hoc t-test: hydrocortisone alone versus yohimbine alone (*p* < .001) and versus placebo (*p* < .001) and the combined group versus placebo (*p* < .001) and versus yohimbine alone (*p* < .001)

** Bonferroni corrected post-hoc t-test: hydrocortisone alone versus the combined group (*p* < .01) and yohimbine alone (*p* < .05) and the combined group versus placebo (*p* < .05)

*** Bonferroni corrected post-hoc t-test: the combined group versus hydrocortisone alone (*p* < .05) and placebo (*p* < .05)
